# Are There Any Different Effects of *Bifidobacterium*, *Lactobacillus* and *Streptococcus* on Intestinal Sensation, Barrier Function and Intestinal Immunity in PI-IBS Mouse Model?

**DOI:** 10.1371/journal.pone.0090153

**Published:** 2014-03-03

**Authors:** Huan Wang, Jing Gong, Wenfeng Wang, Yanqin Long, Xiaochao Fu, Yu Fu, Wei Qian, Xiaohua Hou

**Affiliations:** 1 Division of Gastroenterology, Union Hospital of Tongji Medical College, Huazhong University of Science and Technology, Wuhan, Hubei, China; 2 Division of Gastroenterology, Hospital of Dongfeng Motor Company of Hubei University of Medicine, Shiyan, Hubei, China; 3 Division of Gastroenterology, Sir Run Run Shaw Hospital, School of Medicine, Zhejiang University, Hangzhou, Zhejiang, China; 4 Division of Culture Collection, Hubei Center of Industrial Culture Collection and Research, Wuhan, Hubei, China; Emory University School of Medicine, United States of America

## Abstract

**Background and Aims:**

Research has increasingly suggested that gut flora plays an important role in the development of post-infectious irritable bowel syndrome (PI-IBS). Studies of the curative effect of probiotics for IBS have usually been positive but not always. However, the differences of treatment effects and mechanisms among probiotic stains, or mixture of them, are not clear. In this study, we compared the effects of different probiotics (*Befidobacterium, Lactobacillus, Streptococcus* or mixture of the three) on intestinal sensation, barrier function and intestinal immunity in PI-IBS mouse model.

**Methods:**

PI-IBS model was induced by *Trichinella spiralis* infection in mice. Different probiotics were administered to mice after 8 weeks infection. Visceral sensitivity was measured by scores of abdominal withdrawal reflex (AWR) and the threshold intensity of colorectal distention. Colonic smooth muscle contractile response was assessed by contraction of the longitudinal muscle strips. Plasma diamine oxidase (DAO) and d-lactate were determined by an enzymatic spectrophotometry. Expression of tight junction proteins and cytokines in ileum were measured by Western blotting.

**Results:**

Compared to control mice, PI-IBS mice treated either alone with *Befidobacterium* or *Lactobacillus* (but not *Streptococcus*), or the mixture of the three exhibited not only decreased AWR score and contractile response, but also reduced plasma DAO and D-lactate. These probiotic treatments also suppressed the expression of proinflammatory cytokine IL-6 and IL-17 and promoted the expression of major tight junction proteins claudin-1 and occludin. The mixture of the three probiotic strains performed better than the individual in up-regulating these tight junction proteins and suppressing IL-17 expression.

**Conclusions:**

*Bifidobacterium* and *Lactobacillus*, but not *Streptococcus,* alleviated visceral hypersensitivity and recovered intestinal barrier function as well as inflammation in PI-IBS mouse model, which correlated with an increase of major tight junction proteins. In addition, Mixture of three species was indicated to be superior to a single one.

## Introduction

Irritable bowel syndrome (IBS) is a common functional gastrointestinal disorder usually originated from gut dysfunction with an estimated worldwide prevalence of 10–20% [1], [2], [3]. 7–30% of IBS patients have a history of acute gastrointestinal infection developed post-infectious IBS (PI-IBS)[4], [5]. There are two major pathophysiological findings, including visceral hypersensitivity and dysmotility in the PI-IBS patients. The mechanisms underlying the development of PI-IBS are not fully understood, but are believed to be associate with changes in intestinal permeability and persistent low-grade inflammation[6], [7]. Recently, researches have increasingly suggested that gut flora interacts with the bowel in a complex and dynamic relationship. Therefore, gut flora plays an important role in the induction and progression of PI-IBS[8], [9], [10].

Based on this latter possibility, therapeutic approaches designed to manipulate gut flora with the replenishment of probiotics have been tested in both of patients and animal models with IBS. Several systematic reviews and meta-analyses report that probiotics have a statistically significant effect in improving overall and individual symptoms of IBS patients[11], [12], [13], [14], [15], [16]. Unfortunately, studies of the curative effect of probiotics in IBS have usually, but not always, been positive. Four trails involved with four kinds of *Lactobacillus* fail to show reduction in global symptom score over placebo[17], [18], [19], [20]. Thus, we reasonably conclude that the benefit of probiotics for IBS is likely to be strains specific; nevertheless, comparable studies among species are rare. What’s worse, the study design, probiotic strains and dose are different among studies, which make it difficult to compare the existing results. Which is more effective, single or mixture of species for IBS? Are there any differences among them in the mechanism?

The mechanisms influenced by probiotics that are of potential relevance to the treatment of IBS include enhancement of mucosal permeability, restraining immune activation as well as changes of visceral sensation and motility[21]. Other modes of action, involving enteric neuromuscular modulation and brain-gut axis regulation, are also plausible. In a study of mouse model of PI-IBS, *Lactobacillus paracasei* normalized muscle hypercontractility resulted from a modulation of gut immunologic response to infection[22]. Early life administration of VSL#3, a combination of probiotic strains of various *Bifidobacterium*, *Lactobacillus* and *Streptococcus*, reduces visceral pain perception in a model of IBS[23]. Knowledge of their mechanisms of action is still relatively incomplete. Besides, the results among studies are hard to compare. Can probiotics only be responsible for the changes of the pathophysiologic aspects linked with physiological function of different species in PI-IBS?

In a previous study from our laboratory, NIH mice infected with *Trichinella spiralis* produced alteration in visceral sensitivity, intestinal motility and T helper lymphocytes in lamina propria[24], [25]. These abnormalities persisted after recovery from infection, thus well establishing a model of PI-IBS. To compare the different effects of probiotics, we chose three popular species: *Bifidobacterium longum*, *Lactobacillus acidophilus* and *Streptococcus faecalis*, which were contained by a common probiotic called Bifid Lriple Viable in China and VSL#3 for IBS treatment. We want to investigate the hypothesis that these three stains or their mixture separately change visceral hypersensitivity, contractile hyperresponsiveness, intestinal permeability and inflammation in PI-IBS mouse model.

## Materials and Methods

### Animals

Male NIH mice (6–8 weeks old) were purchased from Medical Animal Laboratory center of Guangdong and kept under specific pathogen-free conditions at Animal Laboratory Center of Tongji Medical College, Huazhong University of Science and Technology. All experiments were approved by the Ethics Committee of Tongji Medical College. (No. 2010–72).

### Trichinella spiralis Infection

*T.spiralis* parasites were obtained from the department of Parasitology at Huazhong University of Science and Technology, Wuhan, China. The colony was maintained through infection among Sprague-Dawley rats. The larvae were obtained from the infected rodents by using a modificated technique described by Castro and Fairbairn[26]. Each mouse was infected by gavaging of 350 *T.spiralis* larvae in 0.2 ml of phosphate-buffered saline (PBS).

### Probiotics Preparation and Administration

Live bacterial strains of *Bifidobacterium longum HB55020* (1.6×10^12^ CFU/g), *Lactobacillus acidophilus HB56003* (2.15×10^11^ CFU/g) and *Streptococcus faecalis HB62001* (3.81×10^12^ CFU/g) were obtained from Hubei Center of Industrial Culture Collection and Research, HBCC. Each strain was mixed with glucose and was converted to freeze dried powder. The mixed powder was packed in sealed bags of 2 g and stored at −20°C for further use.

*T. spiralis*-infected mice after 8 weeks were divided into 5 groups. Each group had 8 mice. Controls were daily gavaged with 0.2 ml PBS for 7 days. The other four groups were separately treated with B*ifidobacterium longum HB55020* (2×10^9^ CFU/d), *Lactobacillus acidophilus HB56003* (1×10^9^ CFU/d), *Streptococcus faecalis HB62001* (0.67×10^9^ CFU/d) and all three probiotics mixture (3.67×10^9^ CFU/d, *Bifidobacterium*: *Lactobacillu*s: *Streptococcus* = 3∶2∶1) for one week.

### Study Design

Visceral sensitivity of each mouse was assessed by behavioral responses to colorectal distention (CRD), which was measured by a semiquantitative score abdominal withdrawal reflex (AWR) and the threshold intensity of CRD that elicits an express contraction in the abdominal wall musculature[25]. Colonic smooth muscle contractile response was studied by measuring the contraction of the longitudinal muscle strips in the organ bath. Plasma diamine oxidase (DAO) activity has been reported to be significantly correlated with lesions and integrity of the intestinal mucosa[27]. D-lactate cumulation in plasma reflects membrane permeability and barrier function of the intestinal mucosa[28], [29]. So plasma DAO activity and D-lactate concentration was used to indirectly evaluate intestinal permeability. The tight junction (TJ) forms a barrier which keeps the apical fluid compartments on opposite sides of the epithelial cell layer and contributes to epithelial paracellular permeability[30]. To explore whether TJ takes effect on intestinal permeability after infection, we analyzed the content of TJ structure proteins in ileum including transmembrane components (claudin-1 and occludin) and cytosolic components (ZO-1) in ileum. Intestinal inflammation was assessed by proinflammatory cytokine profiles of IFN-γ, IL-6 and IL-17. The temporary infection caused by *Trichinella spiralis* mainly occur in the small intestine. Furthermore, gut flora, especially probiotics, becomes more and more from proximal intestine to distal intestine. Based on these facts, we choose terminal ileum to analyse expession of cytokines and tight junction proteins. We studied the parameters that mentioned above in the *T. spiralis*-infected mice after one week treatment or without treatment of *Bifidobacterium longum*, *Lactobacillus acidphilus* and *Streptococcus faecalis*, or mixture of three strains.

### AWR recording to CRD

CRD was performed as described previously [31]. AWR and thresholds were recorded during plastic balloon inflation to 20, 40, 60 and 80 mmHg. AWR score scale was as previously described. The stimulus intensity that evokes a visually identifiable contraction of the abdominal wall was recorded as the threshold intensity of CRD. During the measurements, mice were given CRD for 20 seconds every 4 minutes. To achieve an accurate results, balloon inflation was done 5 times for each value and was observed by two persons.

### Measurement of Contractile Response of Colonic Smooth Muscle to Ach

A piece of mid colon was pinned flat (mucosal side up) in a paraffin-bottomed dissecting dish filled with Krebs solution. Longitudinal muscle strips were taken from each mouse and cut into 3 mm×10 mm pieces and then placed in 25 ml organ bath containing warm (37°C) oxygenated (95%O, 5%CO2) Krebs solution. One end of each strip was attached to an isometric force transducer (Fort-10, WPI, USA) and the other to the armature of the bath. The digitized data were collected by a computer equipped with Acknowledge 3.7.1 software (BIOPAC system, USA). Strips were preloaded with the weight of 1.0 g and allowed to equilibrate in the baths for 60 min with flushing every 20 min. After a stable baseline was attained for 5 min, 10^−5^ mol/L Acetylcholine chloride (Sigma, USA) were added cumulatively to the bath every 5 min. The area under curve (AUC, g·s) was measured at time intervals of 5 min after Ach addition. The response in different groups was quantified by calculating the AUC.

### Measurement of DAO and D-lactate levels

Mouse blood was collected in special centrifugal tube and centrifuged at 3000 bpm for 20 min at 4°C and stored at −80°C. DAO and D-lactate levels were measured by spectrophotometry at 436 nm[32]and enzyme-linked ultraviolet spectrophotometry at 340 nm[33], respectively. O-dianisidine, Gadaverine Dihydrochloride and DAO were purchased from Sigma Chemical Company.

### Western Blot

Ileum tissues were homogenized by mechanical disruption in RIPA buffer with a protease inhibitor cocktail and incubated on ice for 30 min. Lysates were centrifuged at 12000 rpm for 10 min and the protein content of the supernatant was determined by using the BCA protein assay kit. After being diluted with loading buffer and heated to 95°C for 10 min. Depending on the molecular weight, a total of 60 µg of protein lysates derived from ileum tissue samples were loaded onto 8–12% SDS-PAGE gel. Membranes were probed overnight at 4°C with antibodies against tight junction proteins of ZO-1, occludin and claudin-1 (Invitrogen, USA) and cytokines of IFN-γ, IL-17 (R&D, USA) and IL-6 (abcam, USA) or β-actin/GAPDH (Pierce, USA) antibodies, followed by using the appropriate species-specific HRP conjugate (Pierce, USA) and developing in the SuperSignal West Pico Substrate (Pierce, USA). Band intensities were quantified by the Quantity One 4.6.2 software (BioRad, USA).

### Statistical Analysis

AWR scores at each pressure of CRD among the 6 groups were compared using the Kruskal-Wallis one-way analysis of variance on ranks, if the result was significant (*P*<0.05), a Wilcoxon rank sum test with a Bonferroni correction at 0.05/3 to correct for multiple comparisons. Other data were expressed as mean±SEM, and one-way ANOVA was performed among six groups, followed by LSD or DunnettT3 multiple range analysis. A value of *P*<0.05 was considered significant. Statistical analyses were performed with SPSS version 17.

## Result

### Animal model

Morphology: Consistent with previous findings, there were no overt damages of the ileum and colon seen under the microscope after 8 weeks infection. Likewise, the histological scores compared with controls indicated resolution of inflammation.

**Figure 1:**
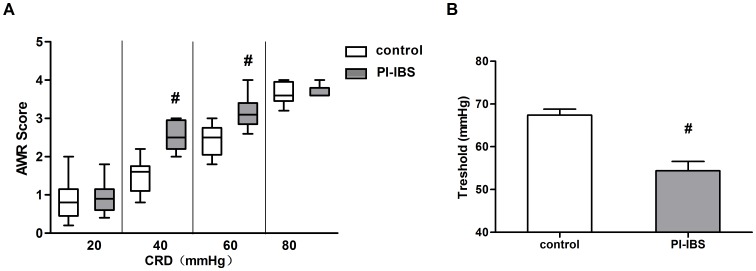
AWR scores and thresholds of control and PI-IBS mouse model. (a) Box plot of AWR scores. Lines represent the median within the box, the 25th and 75th centiles at the ends of the box, and the error bars define the 5th and 95th centiles; n≥8 mice per group. (b) Thresholds of the CRD intensities that evoke abdominal contraction of the mice. Mean±SEM values were plotted; n≥8 mice per group.^ #^p<0.05 PI-IBS group versus control.

Visceral sensation: After infection, mice presented increased visceral sensation contrasted to control (Figure 1). Even when the intestinal inflammation subsided, 8 weeks PI group showed a significant increase of AWR scores for intensities 40, 60 mmHg of CRD, coinciding with lower nociceptive threshold. It suggested that 8week PI group as a good model of PI-IBS with visceral hypersensitivity was managed.

### Effect of Probiotic Strains on Visceral Sensation

**Figure 2:**
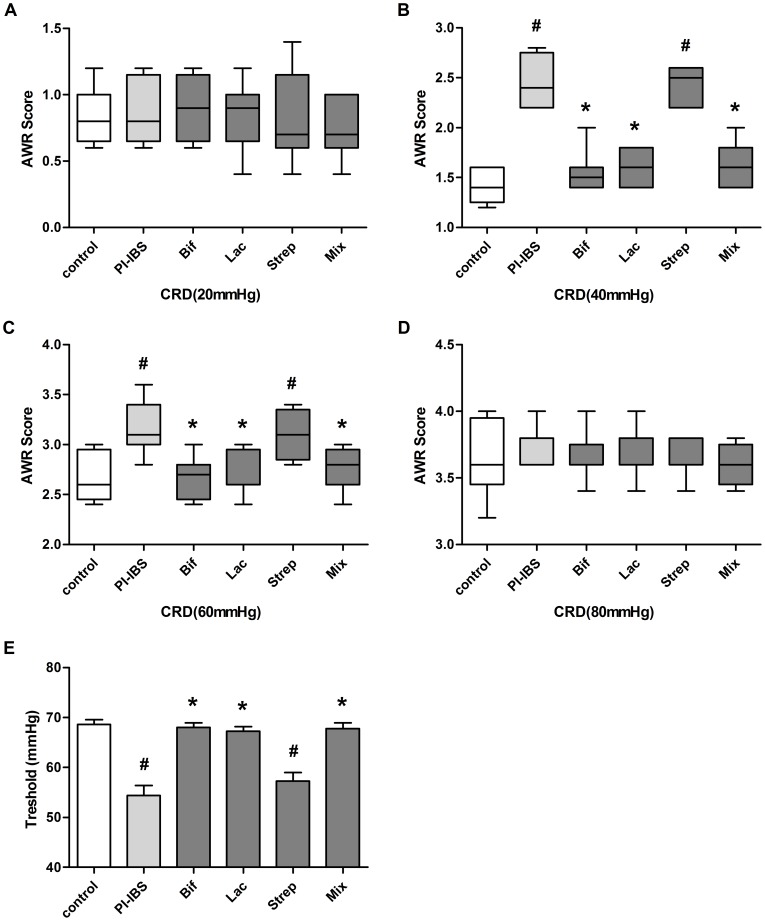
Effect of Probiotic Strains on Visceral Sensation. PI-IBS mice were administered daily with *Bifidobacterium longum*(Bif), *Lactobacillus acidophilus*(Lac), *Streptococcus faecalis* (Strep) or Mixture(Mix) of them for one weeks. Box plot of AWR scores were shown at 20 mmHg(a), 40 mmHg(b), 60 mmHg(c) and 80 mmHg(d). Lines represent the median within the box, the 25th and 75th centiles at the ends of the box, and the error bars define the 5th and 95th centiles; n = 8 mice per group. (d) Thresholds of the CRD intensities. Mean±SEM values were plotted; n = 8 mice per group. ^#^p<0.05 PI-IBS group versus control; *p<0.05 probiotic group versus PI-IBS group.

Except *Streptococcus*, the decrease of visceral hypersensitivity was noted in the *Bifidobacterium*, *Lactobacillus* and Mixture (Figure 2). In the comparison of PI-IBS group, AWR scores of the groups of *Bifidobacterium*, *Lactobacillus* and Mixture obviously decreased at 40 mmHg and 60 mmHg, whereas *Streptococcus* remained higher. Correspondingly, the nociceptive threshold was up regulation in groups of *Bifidobacterium*, *Lactobacillus* and Mixture.

### Effect of Probiotic Strains on Contractile Response of Colonic Smooth Muscle to Ach

**Figure 3:**
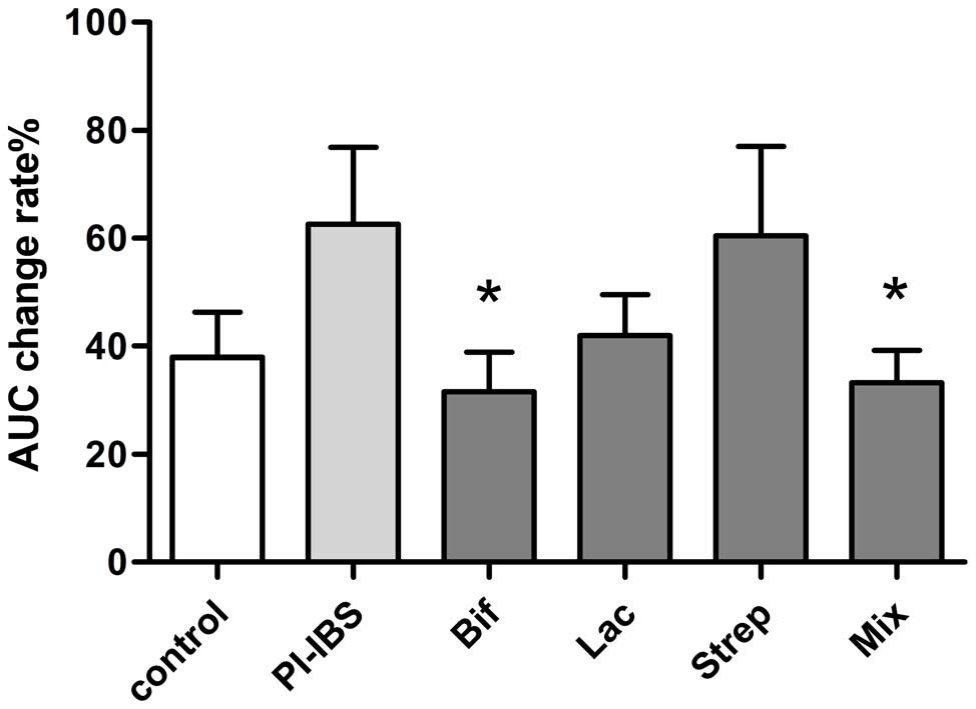
Effect of Probiotic Strains on Contractile Response of Colonic Smooth Muscle to Ach. The AUC change rate of each group was shown by Mean±SEM, n = 8 mice per group. *p<0.05 probiotic group versus PI-IBS group.

Decreasing contractile responses to Ach was observed in longitudinal muscle strips in *Bifidobacterium* and Mixture (Figure 3). Compared with control, PI-IBS mice presented hyperresponsive tendency(p = 0.08) when the response was generated by the longitudinal muscle response to Ach. The change rate of AUC in *Bifidobacterium* and Mixture was significantly decreased over PI-IBS (31.66±7.21vs 62.6±14.25, p = 0.024; 33.31±5.68 vs.62.6±14.25, p = 0.026). However, no difference was found in groups of *Lactobacillus* and *Streptococcus* compared to PI-IBS.

### Effect of Probiotic Strains on intestinal permeability

**Figure 4:**
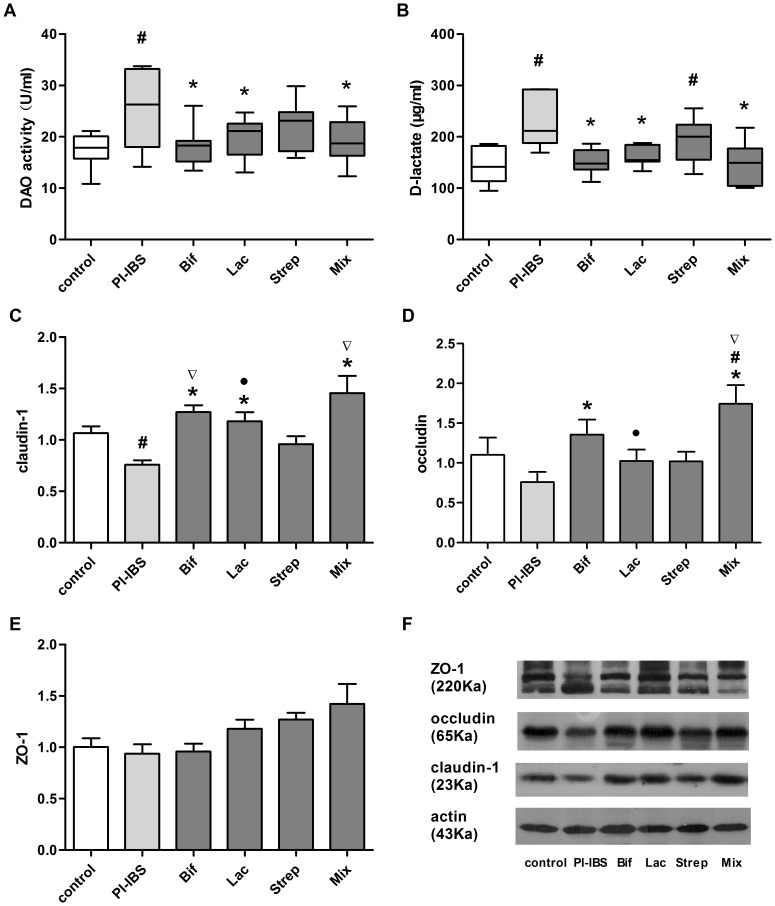
Effect of Probiotic Strains on intestinal permeability. (a) Plasma DAO activity. (b) Pasma D-lactate concentration.Expression of tight junction proteins(c) in ileum: claudin-1, occludin(d) and ZO-1(e). All data were presented by Mean±SEM, n = 5–8 mice per group. ^#^p<0.05 PI-IBS group versus control; *p<0.05 probiotic group versus PI-IBS group; ▽p<0.05 Strep group versus other probiotic groups; •p<0.05 Mix group versus other probiotic groups.

Probiotics could relieve raised intestinal permeability of PI-IBS (Figure 4).

The difference of plasma DAO activity among the groups was statistically significant (p<0.05) (Figure 4a). The mean DAO remained higher in PI-IBS than control (p = 0.004), suggesting that intestinal mucosa injury was persistent even though the histological changes were not obvious. Except *Streptococcus* (22.31±1.80), *Bifidobacterium*, *Lactobacillus* and Mixture showed an evident reduction of plasma DAO activity compared to the group without probiotics given (18.25±1.52, 19.60±1.57 and 19.19±1.85vs25.30±2.90 U/ml). Three effective groups approached control but the *Streptococcus* was still higher. In addition, these three effective groups showed no statistical difference among them.

Similarly, the difference of plasma D-lactate concentration among the groups was statistically significant (p = 0.001) (Figure 4b). The mean D-lactate concentration of PI-IBS and *Streptococcus* group was still higher than control. However, concentration in the groups of *Bifidobacterium*, *Lactobacillus* and Mixture were obviously decreased compared to PI-IBS (151.12±9.51, 163.78±7.92 and 147.88± 17.53vs 232.55±19.84 µg/ml). In contrast with control, no differences were found among the three effective groups. Likewise, there was no statistical difference among the three effective groups.

The results showed that the expression of claudin-1 in terminal ileum was significantly down regulated in PI-IBS group compared with control (0.76±0.04vs1.06±0.07, p = 0.031)(Figure 4c). After administration of probiotics, the groups of *Bifidobacterium*, *Lactobacillus* and Mixture, but not *Streptococcus* group showed an obvious up-regulation in contrast with PI-IBS group. Moreover, three effective groups were in proximity to control and presented no difference among them. Likewise, the difference of expression of occludin among the groups was statistically significant (p = 0.015)(Figure 4d). A lower level of expression of occludin was not noted in PI-IBS group compared with control. However the level of occludin in the *Bifidobacterium* as well as Mixture was higher than PI-IBS (1.35±0.19, 1.74±0.23vs0.85±0.11). Nonetheless, neither *Streptococcus* nor *Lactobacillus* showed statistically difference compared with PI-IBS. Interestingly, the Mixture group was even higher than control. The statistical difference of ZO-1 expression among the groups was not found.

To estimate whether the increased intestinal permeability could have effect on down-regulation of the expression of TJ proteins, the correlation of plasma D-lactate concentration and expression of claudin-1in ileum was analyzed. We found that plasma D-lactate concentration was negatively correlated with the expression of claudin-1 in ileum(r = −0.421, p = 0.004), indicating that up-regulation of the TJ protein by probiotics contributes to recovery of intestinal permeability.

### Effect of Probiotic Strains on cytokine profiles

**Figure 5:**
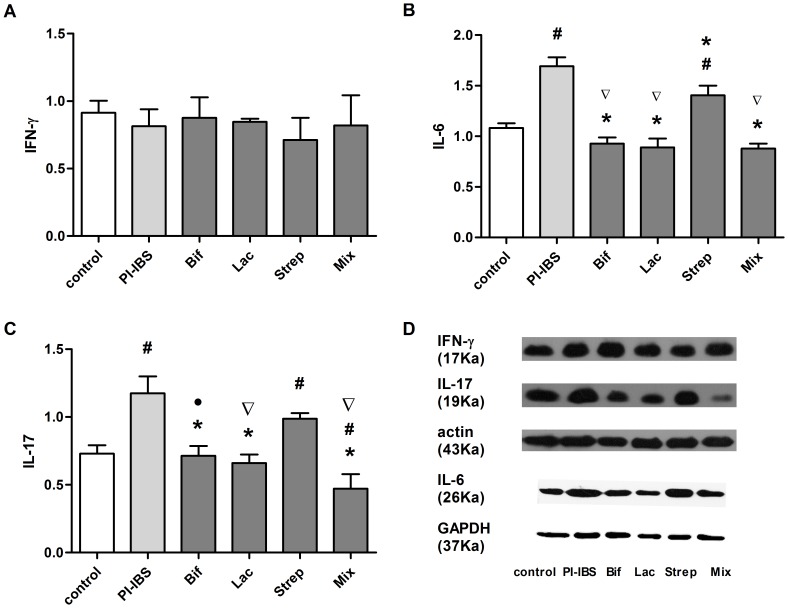
Effect of Probiotic Strains on cytokine profiles. Expression of cytokines(d) in ileum: INF-γ(a), IL-10(d) and IL-17(c). All data were presented by Mean±SEM, n = 5–8 mice per group.^ #^p<0.05 PI-IBS group versus control; *p<0.05 probiotic group versus PI-IBS group; ▽p<0.05 Strep group versus other probiotic groups; •p<0.05 Mix group versus other probiotic groups.

In terminal ileum, IL-6 and IL-17 expression reduced after *Bifidobacterium*, *Lactobacillus* and Mixture administration. But the difference of IFN-γ expression among the groups was not statistically significant. (Figure 5).

In our study, the expression of IL-6 was significantly elevated in PI-IBS group compared with control. Except *Streptococcus* group, the level of IL-6 in the groups of *Bifidobacterium*, *Lactobacillus* and Mixture shared an obvious down-regulation compared to PI-IBS group. Moreover, the level of IL-6 in Mixture was even lower than control (1.08±0.09vs 0.88±0.1).

In comparison with control, IL-17 expression was increased in PI-IBS as well as *Streptococcus* group (p<0.05).The groups of *Bifidobacterium*, *Lactobacillus* and Mixture, but not *Streptococcus* group cut down the expression of IL-17 (0.75±0.07,0.66±0.06, 0.47±0.10vs1.19±0.13). In addition, Mixture was more effective than *Bifidobacterium* (p = 0.02).

### Correlation between intestinal permeability and Visceral Sensation

To explore whether visceral hypersensitivity could be affected by the increased intestinal permeability, the relationship of plasma D-lactate concentration and threshold was analyzed. We found that threshold was negatively correlated with plasma D-lactate concentration (r  = −0.508, p  = 0.001).

## Discussion

In this study, after treatment with *Bifidobacterium*, *Lactobacillus* and Mixture, PI-IBS mouse model presented not only lower AWR scores and contractile response, but also reduction of plasma DAO and D-lactate and cytokines in ileum, suggesting improvement of intestinal hypersensitivity as well as recovery of intestinal barrier function and inflammation. Moreover, our results suggested that probiotic-induced protection of epithelial barrier function may be due to prevention of down-regulation in tight junction proteins expression. However, *Streptococcus* failed to show any favorable effects. What's more notable was that the Mixture of three stains was supposed to be a bit superior to single one.

As described in the results, *Bifidobacterium longum* presented favorable effects, equally with *Lactobacillus*, on sensation, intestinal barrier and inflammation. Nevertheless, *Bifidobacterium* but not *Lactobacillus* reduced contractile hyperresponsiveness to Ach of longitudinal muscle strips. Therefore, *Bifidobacterium longum* was partly superior to other species for treatment of PI-IBS. *Bifidobacterium* is reported to have a great ability to colonize at the intestine, which modify the gut microbiota by producing organic acids such as butyrate acid and competitively adhering to the mucosa and epithelium[34]. Not only does strengthen the gut epithelial barrier, it also modulates the immune system to convey an advantage to the host[35], [36]. As the most commonly used probiotics, *Bifidobacterium* have been extensively studied in IBS[18], [37], [38], [39]. The majority of studies of the therapeutic effect of it in IBS has been positive, indicating mainly beneficial impact on bloating, abdominal pain and flatulence[11]. In particular, a well-designed and frequently quoted trail reveals that *Bifidobacterium infantis 35624*, not *Lactobacillus salivarius UCC4331* significantly improves in abdominal pain/discomfort, bloating/distension and bowel movements compared with placebo[18]. Our result, cionciding with previous study, showed the possible superiority of *Bifidobacterium* for treatment in IBS.

*Lactobacillus acidophilus*, in our study, revealed the improvement of barrier function and reduction of cytokines secretion, thus extending for visceral sensitivity. A lot of studies highlighted the properties of different strains of *Lactobacillus*, mentioning their ability to product the intracolonic short chain fat acid (SCFA) with a consequent improvement in colonic propulsion[40]. However, some of clinical studies are negative and show either no effect or a favorable effect. The divergent results of the efficacy of the *Lactobacillus* used in IBS could be related to different species and doses, suggesting that the effects of *Lactobacillus* may be stains-specific.

Beyond *Bifidobacterium* and *Lactobacillus*, *Streptococcus* has less frequently been used alone in IBS. *Streptococcus faecalis* in this study proved to be ineffective in visceral hypersensitivity, gut permeability and immunomodulatory effects. Although the outcomes of an inactive *Escherichia coli* and *Enterococcus faecalis* bacterial preparation for therapy of IBS have been favorable[41], [42], the overall rationality for their use in IBS has been doubted, because a lack of specific mechanism of action has been confirmed. However, almost all probiotic combinations contained *Streptococcus*, it is therefore possible that *Streptococcus* cooperated with other species of probiotics are synergistic in promoting a therapeutic effect in IBS.

In this study, PI-IBS mouse after gavaged with mixture of three species, ameliorated visceral sense, intestinal permeability and cytokine profiles. Compared with single species, the Mixture has, to some extent, evident advantages. According to the expression of occludin, the Mixture group was higher than *Lactobacillus*. In addition, the Mixture group showed decreased expression of IL-17 compared to *Bifidobacterium*. Based on these results, we could conclude that mixture of three stains was superior to single species. VSL#3, probiotic ‘cocktail’, was reported to be a novel probiotic for the treatment of IBS[43], [44], [45], [46]. In IBS patients with predominant bloating, VSL#3 significantly reduced flatulence scores and retarded colonic transit in contrast to placebo [43]. The comparison between single probiotic and combination probiotic was not reported before, but it turned out that combination was superior to single species in this study. Thus, we have demonstrated the superiority of mixture of three species in barrier protection as well as immunoregulation.

In summary, the literature confirms the benefit of *Bifidobacterium* and *Lactobacillus* alone or the combination of the three species on the gut sensation, intestinal permeability in PI-IBS mouse model; the mechanisms supporting these beneficial effects may be up-regulation of tight junction proteins and restriction inflammation. Nonertheless, *Streptococcus* shows either no effect or a favorable effect. Most importantly, we have demonstrated the superiority of mixture of three species over a single one. This study may aid our understanding of the mechanisms underlying probiotic treatments for PI-IBS, which might offer referrences to select appropriate probitic species for IBS patients with different symptoms.
